# Sleep quality and urinary incontinence in elderly female exercise practitioners

**DOI:** 10.5935/1984-0063.20210003

**Published:** 2022

**Authors:** Débora Verônica da-Luz, Felipe Fank, Franciele da Silva Pereira, Giovana Zarpellon Mazo

**Affiliations:** 1 State University of Santa Catarina, Health and Sport Sciences Center, Department of Physical Education. - Florianópolis - Santa Catarina - Brazil.; 2 State University of Santa Catarina, Health and Sport Sciences Center, Department of Physiotherapy - Florianópolis - Santa Catarina - Brazil.

**Keywords:** Sleep, Older adults, Urinary incontinence, Nocturia, Exercise

## Abstract

**Objectives:**

Older women with urinary incontinence (UI) commonly report sleep problems. However, little is known about sleep problems in older women with UI who exercise. We aimed to evaluate the relationship of sleep quality with the presence or absence of UI, urine loss, and nocturia episodes in elderly female practitioners of physical exercise.

**Material and Methods:**

We conducted a cross-sectional study on older women participating in an extension program, southern Brazil. Data were collected in 2018. Sociodemographic and health characteristics were collected by interview. The International Consultation on Incontinence Questionnaire - short form was applied to evaluate the presence of UI, as well as the frequency and amount of urine loss. The International Consultation on Incontinence Questionnaire Overactive Bladder was used to analyze nocturia episodes. Sleep quality was assessed using the Pittsburgh sleep quality index.

**Results:**

The study group included 142 older women with a mean age of 68.8±6.8 years. The prevalence of UI was 33.8% (95% CI: 0.26-0.42). Women with UI had higher use of medication, daytime dysfunction, and total sleep quality scores than women without UI (p<0.05). Older women who leak urine several times a day exhibited worse sleep quality than those who lose urine once a week or less (p=0.036). Women with three or more episodes of nocturia also showed worse sleep quality than those without any episode (p=0.029). **Conclusion:** Exercising older women with UI have worse sleep quality than those without this dysfunction. Furthermore, participants who lose large amounts of urine and have more episodes of nocturia also exhibit worse sleep quality.

## INTRODUCTION

Urinary incontinence (UI) - defined as any complaint of involuntary loss of urine^1^ - is considered a geriatric syndrome^2^. This dysfunction can affect women of different ages, although a higher prevalence is observed among older women^3^, which can reach 52.2% in this population^4^. Additionally, UI is considered a predictor of death and this risk increases with increasing severity of this condition^5^.

In addition to being intimately associated with aging, UI is also related to sleep quality^6^. Sleep is a physiological process that is fundamental for life^7^. The prevalence of poor sleep quality is high among women with UI^8^ and some symptoms associated with this dysfunction also affect the sleep of women, particularly nocturia^9^. Non-pharmacological therapies have been proposed for symptom improvement in both cases. More specifically, physical exercise appears to be an effective tool to improve the symptoms of UI^10^, as well as to reduce sleep disorders and to improve the quality and duration of sleep^11^.

In older women, exercise improves the overall quality of sleep and reduces nocturnal fluctuations through physiological, biochemical, and psychological changes^12^. In a systematic review with meta-analysis, Hwang and Shin (2016)^13^ identified exercise as the non-pharmacological intervention with the greatest positive effect on insomnia. With respect to UI, a systematic review demonstrated that regular physical activity is a protective factor against this dysfunction, while older women with a sedentary lifestyle had an increased risk of developing UI^3^. However, there is a lack of studies evaluating the prevalence of UI among elderly female exercise practitioners and the relationship of this dysfunction with sleep quality in this specific population. In a systematic review on the prevalence of UI in older women^14^, only one of the 11 articles included in the study analyzed elderly exercise practitioners; however, none of them investigated sleep.

Furthermore, it is important to investigate whether sleep quality is related to the severity of UI, especially in regularly exercising older women, since 33% of this population experience involuntary losses of urine during the activity^15^. Therefore, the present study aimed to evaluate the relationship of sleep quality with the presence or absence of UI, urine loss, and episodes of nocturia in elderly female practitioners of physical exercise.

## MATERIAL AND METHODS

### Study design and ethical aspects

This cross-sectional, observational study was approved by the Ethics Committee on Research Involving Humans of the State University of Santa Catarina (UDESC) (ethical clearance certificate 4588115.1.000.0118). All participants signed the free informed consent form in accordance with Resolution N. 466/2012 of the National Health Council. The study was conducted according to the STROBE (strengthening the reporting of observational studies in epidemiology) recommendations.

### Participants

Older women enrolled in the physical exercise program of the extension study group for older people (GETI) of UDESC participated in the study. A consecutive sample was obtained by convenience sampling. One hundred and forty-six older women participated in GETI in 2018. Of these, 142 agreed to participate in the study and answered the questionnaires. The criteria for inclusion in the study were female sex, age of 60 years or older, and participation in one of the exercise modalities for at least 6 months. Older women who did not come to the interview on the day scheduled for data collection for any reason and those who did not respond to all questionnaires were excluded from the study. The exercise modalities of GETI include walking, gymnastics, water aerobics, swimming, weight training, and Pilates, offered twice a week, with each session lasting 50 minutes.

### Variables and instruments

#### Sociodemographic characteristics, health conditions, and anthropometric data

A diagnostic form was applied to collect the age, sociodemographic characteristics (marital status and educational level), and health conditions (health perception, diseases, and medication use) of the participants. Health perception was collected by asking the participants how they perceived their current health status: very bad, bad, regular, good, and very good. The responses were then categorized as positive (good and very good) and negative (very bad, bad, and regular). Diseases were investigated by asking the older women if they had any disease diagnosed by a doctor. Medication use was evaluated by asking the participants whether they used any medication.

The body weight (in kg) was measured with a digital scale (Millenium Prata CA6000, G-Life^®^, 150kg). Height (in meter) was measured with a Cardiomed^®^ stadiometer (height limit of 2.16m) at the highest point of the head. The anthropometric variables were measured twice and the mean of the two measurements was considered for analysis. In the case of a large difference between the two measurements, a third one was obtained and considered the final result. The anthropometric data were used to calculate the body mass index (BMI) as the ratio of body weight to height squared (kg/m^2^).

### Urinary incontinence

The International Consultation on Incontinence Questionnaire - Short Form (ICIQ-SF) was used to evaluate UI. This instrument was developed by Avery et al. (2004)^16^ to assess the impact of UI on quality of life and to evaluate urine loss in patients of both sexes. In addition, this questionnaire measures the frequency and amount of urine loss and to what extent it interferes with everyday life of the respondents. The following question was used in this study to determine the presence of UI: “Do you currently leak urine?”.

### Nocturia

The International Consultation on Incontinence Questionnaire Overactive Bladder (ICIQ-OAB) was used to evaluate episodes of nocturia in the older women. This instrument assesses the impact of a hyperactive bladder on quality of life by investigating urinary frequency, urinary urgency, and symptoms of nocturia^17^. The following question was used in the present study: “How often, on average, do you need to wake up during the night to urinate?”. Possible answers were none, one or two, or more than three episodes per night.

### Sleep quality

The Sleep Quality of the Participating Older women was assessed using the Pittsburgh sleep quality index (PSQI), validated to use in Brazil^18^. The PSQI consists of 19 questions that evaluate different sleep-related problems during the last month. The questions are divided into seven components and each question is scored from 0 to 3. The components are sleep quality, sleep duration, sleep latency, habitual efficiency, sleep disturbances, use of sleep medication, and daytime dysfunction. The sum of scores for each component generates a total score ranging from 0 to 21, with a higher score indicating worse sleep quality. A total score higher than 5 indicates poor sleep quality, while lower scores indicate good sleep quality.

### Data collection

Data were collected in November 2018. After the older women had agreed to participate in the study, the day and time for application of the questionnaires were scheduled. Previously trained researchers applied the instruments through individual interview on the premises of the university. All interviews took place in a quiet room to facilitate understanding by the older women and to make them more comfortable to answer. The instruments were applied in the following order: 1) diagnostic form; 2) anthropometric measurements; 3) ICIQ-SF; 4) ICIQ-OAB; and 5) PSQI.

### Statistical analysis

The data were entered into the *Excel*^®^ program and the IBM SPSS 20.0 software was used for the analyses. The sociodemographic and health characteristics were analyzed using descriptive statistics (absolute and relative frequency). Age and PSQI component and total scores are reported as mean and standard deviation.

The normality of the data was first verified by the Kolmogorov-Smirnov test (total sample), and then by the Shapiro-Wilk test (only older women with UI). The Mann-Whitney U test was applied to evaluate differences in mean PSQI component scores between older women with and without UI. The independent t-test was used for the same analysis but considering the total PSQI score. The frequency and severity of urine loss and nocturia episodes were compared according to total PSQI score using one-way ANOVA with Tukey’s post-hoc test to determine in which categories the difference occurred. A level of significance of 5% was adopted for all tests.

## RESULTS

One hundred and forty-two women with a mean age of 68.8±6.82 years participated in the study. Most of the participants were married (45.3%) and 38.0% had complete elementary school. Regarding health characteristics, 67.9% of the sample were classified as normal weight according to BMI, 66.4% reported to have a positive health perception, 87.6% had one or more diseases, and 81.0% reported to use medications. The prevalence of UI in the sample was 33.8% (95% CI: 0.26-0.42). Comparison of older women with and without UI showed a tendency of separated/divorced women and those with positive health perception not to have UI. The detailed data are presented in Table 1.

**Table 1:** Sociodemographic and health characteristics of the study sample.

Variable	Total (n=142)	With UI (n=48)	Without UI (n=94)	*p*-value
**Age^(^X^/SD)^**	68.8 (6.8)	69.7 (6.6)	68.4 (6.9)	0.116^¥^
**Marital status (^n/%)^**				
Married	62 (45.3)	25 (54.3)	37 (40.7)	0.042^€^**
Separated/divorced*	26 (19.0)	3 (6.6)	23 (25.2)	
Single	9 (6.6)	4 (8.7)	5 (5.5)	
Widowed	40 (29.1)	14 (30.4)	26 (28.6)	
**Education^(f/%^)**				
Without education	33 (24.2)	15 (32.6)	18 (19.8)	0.156^€^
1 to 8 years of schooling	65 (47.4)	23 (50.0)	42 (46.2)	
9 to 11 years of schooling	24 (24.8)	7 (15.2)	27 (29.7)	
Higher education	5 (3.6)	1 (2.2)	4 (4.3)	
**Health perception^(f/%)^**				
Positive	91 (66.4)	25 (54.3)	66 (72.5)	0.037£**
Negative	46 (33.6)	21 (45.7)	25 (27.5)	
**Diseases^(f/%)^**				
Yes	120 (87.6)	42 (91.3)	78 (85.7)	0.421^£^
No	17 (12.4)	4 (8.7)	13 (14.3)	
**Medication use^(f/%)^**				
Yes	111 (81.0)	40 (87.0)	71 (78.0)	0.253^£^
No	26 (19.0)	6 (13.0)	20 (22.0)	
**BMI^(f/%)^**				
Underweight	9 (6.6)	4 (8.9)	5 (5.4)	0.197^€^
Normal range	93 (67.9)	26 (57.8)	67 (72.8)	
Overweight	35 (25.5)	15 (33.3)	20 (21.8)	

**Notes:** = X Mean; SD = Standard deviation; UI = Urinary incontinence; f = Frequency; BMI = Body mass index;

¥ Mann-Whitney U test;^€^Fisher’s exact test;

£ Chi-square test;

* Difference between groups;

** Significant difference.

Table 2 shows the comparison of sleep quality and its components between older women with and without UI. A significant difference was observed for the total score (*p*=0.028) and for the sleep medication (*p*=0.049), and daytime dysfunction components (*p*=0.028). Older women with UI had a higher mean total score (6.71±4.21 vs. 5.19±3.66) and higher medication (0.71±1.20 vs. 0.34±0.85) and daytime dysfunction scores (0.73±0.84 vs. 0.41±0.61).

**Table 2:** Components of PSQI according to the presence or absence of urinary incontinence in older women who exercise (n=142).

Components of PSQI	Urinary incontinence		
	**With (n=48)**	**Without (n=94)**	*p-* **value^#^**
	X **(SD)**	X **(SD)**	
Sleep quality	1.00 (0.55)	0.89 (0.63)	0.212
Sleep latency	1.33 (1.19)	1.03 (1.11)	0.145
Sleep duration	1.13 (0.98)	0.87 (0.91)	0.129
Habitual sleep efficiency	0.79 (1.05)	0.73 (1.11)	0.470
Sleep disorders	1.02 (0.53)	0.90 (0.49)	0.196
Use of medication	0.71 (1.20)	0.34 (0.85)	0.049*
Daytime dysfunction	0.73 (0.84)	0.41 (0.61)	0.028*
Global PSQI score	6.71 (4.21)	5.19 (3.66)	0.028*

**Notes:** PSQI = Pittsburgh sleep quality index; X = Mean; SD = Standard deviation;

* Significant difference;

# Mann-Whitney U test.

Table 3 shows the comparison of total PSQI scores according to severity of urine loss and nocturia episodes among women with UI. The results showed that the total PSQI score differed significantly according to the frequency of urine loss (*p=*0.036) and episodes of nocturia (*p*=0.029). Older women who reported to leak urine several times a day had worse sleep quality (10.80±5.63) than those who leak urine once a week or less (5.52±3.48). Older women who need to wake three times or more during the night to urinate had worse sleep quality (9.07±4.98) than those who do not need to urinate at night (5.57±3.78). Sleep quality did not differ according to the amount of urine loss, although older women reporting to lose a large amount of urine had a higher sleep quality score than those who lose a moderate or small amount.

**Table 3:** Comparison of total PSQI scores according to severity of urine loss and nocturia episodes among women with UI (n=48).

Variable	Total PSQI score	*p*-value^#^
	X **±SD**	
**Frequency of urine loss**		
Once a week or less^¥^	5.52±3.48	0.036*
Two or three times a week	6.29±3.43	
Once a day	8.83±5.35	
Several times a day^¥^	10.80±5.63	
**Amount of urine loss**		
A small amount	6.39±3.92	0.146
A moderate amount	6.43±3.82	
A large amount	11.33±4.21	
**Episodes of nocturia**		
None^¥^	5.57±3.78	0.029*
One or two	5.68±3.16	
Three or more^¥^	9.07±4.98	

**Notes:** = X Mean; SD = Standard deviation;

¥ = Difference between groups;

* Significant difference;

# One-way ANOVA with Tukey’s post-hoc test.

## DISCUSSION

The aim of the present study was to investigate the relationship of sleep quality with the presence or absence of UI, urine loss, and episodes of nocturia in elderly female exercise practitioners. Older women with UI had higher mean medication use and daytime dysfunction component scores and a higher total PSQI score than those without UI. In addition, the higher the frequency of urine loss and episodes of nocturia, the worse was the sleep quality of older women with UI.

The prevalence of older women with UI in this study was considered high and was similar to that found in other studies involving this population^4,19^. However, the literature reports wide variability in the prevalence of UI among women. This variation might be due to differences in the characteristics of the sample such as age and place of study and in the type of instrument used for the assessment of UI, as well as methodological differences between the studies^20^.

In the present study, older women with UI had worse sleep quality than participants without the dysfunction. Urinary incontinence is a geriatric problem that can cause sleep disorders and these two variables are associated^6^. A study involving 645 older women demonstrated that 58% of the participants with urgency UI had poor sleep quality, providing new evidence that the degree of the sleep disorder is correlated with the severity of UI^8^. In addition, older adults with UI are more likely to develop sleep problems than those without the dysfunction (OR=4.03; 95% CI: 1.74-9.25)^6^.

Nocturia is one condition that can explain the relationship between sleep quality and UI. Nocturia, which is defined as the need to wake up during the night to urinate, is one of the most common reasons for waking up at night^21^ and has been shown to be correlated with poor sleep quality^22^. In our study, older women who woke up more than three times per night had worse sleep quality than those who did not wake up at night to urinate. This result is consistent with the literature and clearly demonstrates the relationship between sleep quality and UI.

We also observed that older women who reported to leak urine several times a day had worse sleep quality than those with a frequency of urine loss of once a week or less. The severity of urine loss seems to be proportional to sleep quality^8^, i.e., the higher the frequency of loss, the higher the PSQI score and consequently the poorer the sleep quality. To understand this relationship, it is necessary to analyze overall sleep quality and not only nighttime sleep. For example, in the present study, older women with UI had greater daytime dysfunction - a component of the PSQI - than those without UI. This finding shows that UI does not only impact nighttime sleep but also the daytime period in older women.

Older women with UI also exhibited a higher average medication use than those without UI. Most participants reported the daily use of at least one medication, a finding that might be related to the presence of UI. One study^23^ showed that pharmacological treatment of UI improved related symptoms as well as the participants’ sleep quality, demonstrating the relationship between medication use and these two variables. Another study found an association between the development of UI and the use of sleep medications^24^. Furthermore, the use of some types of drugs such as adrenergic blockers, diuretics, angiotensin-converting enzyme inhibitors, and benzodiazepines might be associated with transient UI^25^.

In the present study, all older women regularly exercised. Several systematic reviews indicate exercise as a very effective non-pharmacological treatment to improve sleep quality and related variables^26,27^. Within this context, physical activity/exercise seems to be related to better sleep quality, as indicated by the observation of a higher prevalence of sleep disorders in individuals with low physical activity levels^28^. One study involving older women concluded that nocturnal variations in sleep efficiency are influenced by exercise, which can improve both overall sleep quality and nocturnal alterations^12^. Therefore, our results may have been influenced by the fact that the older women regularly exercise. In a sample of sedentary or insufficiently active women, sleep quality may become even worse with increasing severity of UI.

Regarding exercise intensity, vigorous exercise is associated with urine loss because of impairment of the support, suspension and restrain mechanisms of the pelvic muscles, which suffer intense and repeated overload that weakens the pelvic floor^29^. Thus, exercises of moderate intensity are recommended, which are a protective factor against UI^3^. In addition, pelvic floor muscle training is used as an exercise strategy to strengthen the pelvic floor muscles and to reduce the number of episodes of urine leakage in women with different types of UI^1^.

This study has some limitations. The PSQI has not been validated for older adults. In addition, UI was assessed using indirect instruments (ICIQ-SF and ICIQ-OAB). Thus, new studies using direct measures such as the Peritron are recommended. Furthermore, the study did not evaluate medications used specifically for sleep disorders or UI. Also, cross-sectional studies cannot be used to infer causality; experimental and follow-up studies should therefore be carried out to identify the relationship more clearly between UI, sleep, and exercise. Finally, the objective of our study was to verify whether the elderly’s sleep was affected by the severity of UI. Thus, we only made comparisons. Also, the number of participants in this study limits the performance of more robust association analysis, such as binary regression. Despite these limitations, this is the first study to analyze sleep quality related to UI in regularly exercising older women.

## CONCLUSION

The present study showed that elderly female exercise practitioners with UI had worse sleep quality than those without the dysfunction. Older women with UI also had higher mean medication and daytime dysfunction component scores. The total PSQI score was higher in older women who leak urine several times a day and women with three or more episodes of nocturia when compared to those without UI. However, no difference was found when sleep quality was compared according to the amount of urine loss, although women losing large amounts had a higher PSQI score.

Alterations in sleep quality occur with aging and sleep quality may be influenced by the presence of UI. Thus, regular physical exercise could be a strategy to attenuate these dysfunctions since the prevalence of sleep problems is known to be higher in individuals with low physical activity levels. We therefore suggest new studies that analyze sleep quality related to UI in active older women using direct assessment methods. This study may also encourage health professionals to direct efforts towards the prevention of UI and sleep disorders in older women through self-care strategies and the promotion of physical exercise.
